# Intraparenchymal Hematoma With Significant Mass Effect Treated With Factor Eight Inhibitor Bypass Activity

**DOI:** 10.7759/cureus.31385

**Published:** 2022-11-11

**Authors:** Sathwika Prasad, Paola Colon Figueroa, Thor S Stead, Rohan K Mangal, Latha Ganti

**Affiliations:** 1 Emergency Medicine, University of Central Florida College of Medicine, Orlando, USA; 2 Neurology, University of Central Florida College of Medicine, Orlando, USA; 3 Medicine, The Warren Alpert Medical School of Brown University, Providence, USA; 4 Medicine, University of Miami Miller School of Medicine, Miami, USA; 5 Emergency Medicine, HCA Florida Ocala Hospital, Ocala, USA; 6 Emergency Medicine, Envision Physician Services, Plantation, USA

**Keywords:** factor eight inhibitor bypass activity, prothrombin complex concentrate, andexanet alfa, fresh frozen plasma, intraparenchymal hematoma

## Abstract

The authors present the case of an 80-year-old female with myelodysplastic syndrome treated with chemotherapy and apixaban, a direct oral anticoagulant who suffered an intracranial hemorrhage. She presented to the emergency department with altered mental status and was found to have a large intraparenchymal hematoma with a significant mass effect. Our patient was given FEIBA (Factor Eight Inhibitor Bypass Activity) to reverse the hemorrhage. Anticoagulant-related bleeding reversal strategies are discussed.

## Introduction

Intracerebral hemorrhage (ICH), of which intraparenchymal hematomas are a subtype, makes up less than 20% of all stroke cases but is associated with early-term mortality of 30% to 40% [1]. The mortality remains high despite decreasing incidence [2]. Amongst survivors, less than 50% are able to return to their previous level of activity.

Risk factors for ICH include prior ICH, hypertension, hyperlipidemia, diabetes, metabolic syndrome, myeloid angiopathy, and many medications. Drugs that increase the risk of ICH include vasoconstrictive agents such as triptans and selective serotonin re-uptake inhibitors, decongestants, stimulants, phentermine, and sympathomimetic drugs. Estrogen-containing oral contraceptives can result in hemorrhage attributable to cerebral venous sinus thrombosis. Furthermore, drugs of abuse such as tobacco, marijuana, cocaine, alcohol, and amphetamines also increase the risk of ICH [3].

The traditional presentation of ICH is a sudden onset of focal neurological deficits, which progress rapidly over the course of minutes to hours [4]. The deficits are often accompanied by nausea, headache, decreased consciousness, and elevated blood pressure.

The main goals in the emergent management of ICH are to prevent further bleeding, and assess whether surgical intervention is of benefit. Towards this end, the focus is on blood pressure control and agents directed at reversing any underlying coagulopathy. Our patient was on a direct oral anticoagulant (DOAC) for her myelodysplastic syndrome and was given FEIBA (Factor Eight Inhibitor Bypass Activity) to reverse the hemorrhage. 

## Case presentation

An 80-year-old female presented to the emergency department (ED) with a deteriorating mental status. The patient’s son stated that she had full cognitive function the previous night. She started feeling sick the morning of the ED presentation and vomited a few times. A few hours later, she was unable to drink water with a straw, and could only answer questions with “gibberish.” Her symptoms evolved until she could no longer open her eyes or talk. Her past medical history was significant for a myelodysplastic syndrome which was being treated with chemotherapy and apixaban, a DOAC. She had taken her medication within the previous 12 hours. Her son stated she did not smoke or use any recreational drugs or alcohol.

Her vital signs included a temperature of 98.5°F, blood pressure of 138/81 mmHg, a pulse of 100 beats per minute, a respiration rate of 16 breaths per minute, and an oxygen saturation of 96% on room air. Physical examination showed multiple areas of ecchymosis of different ages, including periorbital ecchymosis, but no eye injury. She also had scattered petechiae. The patient only opened her eyes to noxious stimuli and moaned a few times. She was, however, able to protect her airway. Her Glasgow coma score was a 9 (E2V2M5). Her cardiopulmonary and abdominal exams were unremarkable. Laboratory analysis was significant for severe thrombocytopenia, anemia, hyperglycemia, mild hypokalemia, and hyponatremia (Table 1).

**Table 1:** Patient's laboratory analysis reports POC = point of care; ABG= arterial blood gas; pCO2 = partial pressure of carbon dioxide; pO2= partial pressure of oxygen; CO2= carbon dioxide; O2= oxygen; HCO3= bicarbonate

	Patient result	Reference range (hospital standard)
Blood Gas		
POC ABG pH	7.42	(7.35 - 7.45)
POC ABG pCO2	25.3 L	(35 - 45 mmHg)
POC ABG pO2	81	(80 - 105 mmHg)
POC ABG HCO3	16.3 L	(22-26 mmol/L)
ABG Total CO2	17 L	(23 - 27 mmol/L)
ABG O2 Saturation	96%	(95-98%)
POC ABG Base Excess	-7 L	(-2-3 mmol/L)
Chemistry		
Sodium	134 L	136 - 145 mmol/L
Potassium	3.6 L	3.7 - 5.1 mmol/l
Chloride	97 L	98 - 107 mmol/L
Carbon Dioxide	18 L	21 - 32 mmol/l
Anion Gap	22.6	7 - 18 mg/dl
Blood Urea Nitrogen	25 H	0.55 - 1.3 mg/dl
Creatinine	1.21	0.6 - 1.3 mg/dL
Glucose	267 H	74 - 106 mg/dl
Calcium	9.2	8.4 - 10.1 mg/dl
Total Bilirubin	1.4	0.2 - 1.5 mg/dl
Aspartate Transaminase	11	10 - 37 unit/L
Alanine Aminotransferase	8 L	12 - 78 unit/L
Total Alkaline Phosphatase	61	45 - 117 unit/L
Creatine Kinase	40	21 - 215 unit/L
Total Protein	7	6.4 - 8.2 g/dL
Albumin	3.1 L	3.4 - 5.0 g/dl
Coagulation Studies		
Prothrombin Time	13.3 H	9.0 - 12.7 seconds
International Normalized Ratio	1.3 H	0.85 - 1.17
Hematology		
White Blood Cell Count	2.13 L	4.0 - 12.0 K/mm^3^
Hemoglobin	6.3 C	12.0 - 16.0 gm/dL
Hematocrit	19.0 C	37.0 - 47.0 %
Platelet Count	1 C	130 - 400 K/mm^3^
Immature Granulocytes %	1 H	0 - 0.22 %
Neutrophils %	52.1	38.0 - 74.0 %
Lymphocytes %	40.8	20 - 45 %
Monocytes %	6.6	3 - 10 %
Eosinophils %	0	0 - 4 %
Basophils %	0	0 - 2.0 %

**Figure 1:**
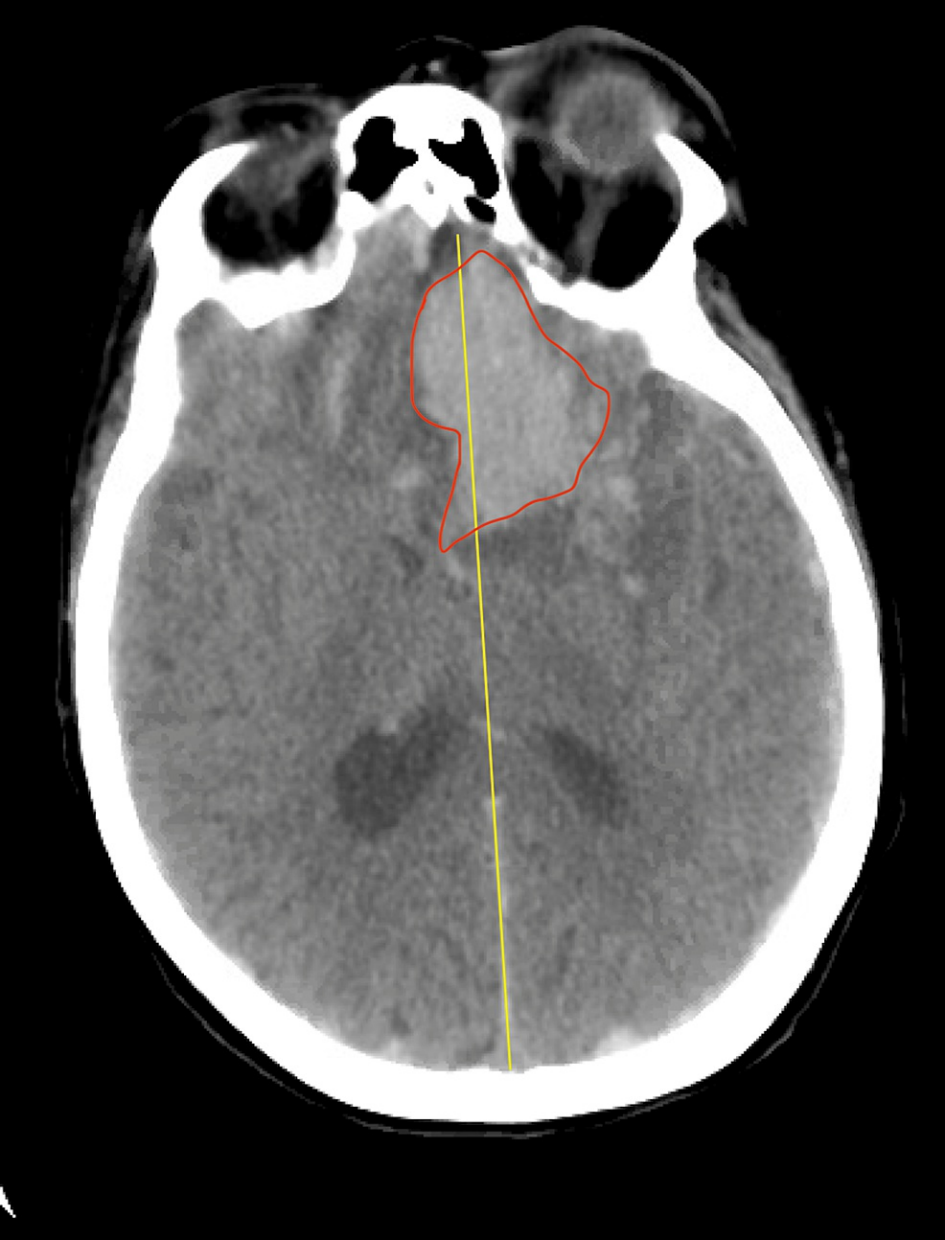
Patient's non-contrast CT scan The CT scan image is demonstrating a large intraparenchymal hematoma (red outline) and  shift away from the midline (yellow line)

Noncontrast brain CT demonstrated a large left frontal intraparenchymal hematoma with significant mass effect, resulting from a midline shift anteriorly left to right of approximately 11 mm. Also present was extra-axial hemorrhage in the parafalcine region as well as anterior to the bilateral frontal lobes and in the basal cisterns. Additionally, there was an intraventricular extension of hemorrhage, relative effacement of the left lateral ventricle, and hemorrhage in the occipital horns of the lateral ventricles bilaterally. The left parietal parenchyma demonstrated a rounded focus approximately 1.4 cm in diameter, and a small volume subarachnoid hemorrhage is seen in close proximity at the left parietal cortex (Figure 1).

Her ICH score was a 4, with 1 point each for age 80, GCS between 5-12, ICH volume > 30 cubic centimeters, and the presence of intraventricular blood. The patient was given FEIBA for reversal of anticoagulation and a platelet transfusion. An ICH score of 4 is generally associated with a 97% mortality. Given her poor prognosis, the family opted for hospice care.

## Discussion

Coagulopathy-associated ICH is an additional risk factor for mortality and poor functional outcome with hematoma expansion which may occur within one hour in up to 25% of patients and within four hours in 88% of patients. Anticoagulant-related ICH is a medical emergency that requires emergent reversal [5]. Oral anticoagulation use is increasing due to the aging population and the associated increase in patients with cardiovascular comorbidities. There are several reversal agents available, with availability varying by the institution. Current anticoagulation options include vitamin K antagonists (VKAs) such as Warfarin and DOACs. DOACs are divided into two classes; direct thrombin inhibitors and factor Xa inhibitors. Treatment should be initiated based on neuroimaging and clinical information such as type and time of anticoagulant dosing rather than waiting for laboratory results [3].

Warfarin interferes with the production of vitamin K-dependent clotting factors II, VII, IX, and X by depleting vitamin K reserve [5]. Warfarin-related coagulopathy is usually treated with Prothrombin Complex Concentrate (PCC) which corrects coagulopathy by replacing all four vitamin K-dependent factors. It can be administered in a rapid small volume for which it is considered the preferred treatment. Fresh Frozen Plasma (FFP) is derived from whole blood products and works by replacing plasma proteins with replete clotting factors. FFP is ineffective in the acute setting because reversal of INR (international normalized ratio) with FFP may take up to 24 hours. Additionally, FFP requires high volumes and can worsen fluid balance which may lead to pulmonary edema in patients with heart failure.

Heparin-induced coagulopathy is treated with protamine sulfate. Dosage may vary depending on the duration of the last dose and the route; subcutaneous or intravenous route [6]. DOACs have been shown to be safer than warfarin in terms of risk of stroke or systemic embolism, intracranial bleeding, and all-cause death compared with warfarin [7]. Given their safety, efficacy, and ease of use, their use has been increasing.

Andexanet alfa is a reversal agent for factor Xa inhibitors and is currently the only FDA-approved agent for apixaban and rivaroxaban. The single-arm ANNEXA-4 study reported 82% of patients had excellent or good hemostatic efficacy at 12 hours, after the administration of andexanet alfa [8]. Given its cost and limited availability, PCC is still commonly used. There is insufficient evidence about risks and benefits to strongly favor either 4-factor PCC or andexanet over the other; these reversal agents have not been compared with each other directly in a randomized trial. Data comparing outcomes in patients given either andexanet alfa or PCC are limited by baseline imbalances between the groups attributable to selection bias [3].

Factor VIII inhibitor bypassing activity (FEIBA), an activated prothrombin protein complex concentrate, is also used as an off-label factor Xa inhibitor reversal agent. It controls bleeding by induction and facilitation of thrombin generation [9]. A few studies report good hemostasis with FEIBA in DOAC-associated bleeding. A case series of 104 patients that used FEIBA specifically for bleeding secondary to apixaban or rivaroxaban obtained hemostasis in 89% of patients [10].

A study of 64 consecutive patients on a DOAC found both low-dose FEIBA (<20 units/kg) and moderate doses (20-30 units/kg) to be an effective management strategy for obtaining hemostasis in DOAC-related ICH [11]. Despite a handful of studies reporting that FEIBA is a safe and effective agent for the reversal of apixaban and rivaroxaban, it is still considered off-label for DOAC-associated bleeding at the time of this writing. 

## Conclusions

There are multiple ways to manage coagulopathy-associated ICH, such as PCC, andexanet alfa, and FEIBA. Overall, there has been significant progress in the options for the management of ICH in patients on oral anticoagulation. Failure to address coagulopathy may result in the delay of neurosurgical intervention, an increase in the size of the hematoma, and an increased risk of mortality.
